# Intramedullary Hemangioblastoma in the Cervical Spinal Cord of a Dog

**DOI:** 10.1155/crve/9197786

**Published:** 2025-10-19

**Authors:** Kiyotaka Arai, Haruto Kushige, Osamu Sakai, Ryohei Yoshitake, Kenji Kutara, Keisuke Sugimoto, Natsuki Akashi, Shinichi Nakamura, Akihiko Sugiyama

**Affiliations:** Faculty of Veterinary Medicine, Okayama University of Science, Imabari, Japan

**Keywords:** hemangioblastoma, immunohistochemistry, intramedullary, magnetic resonance imaging, spinal cord

## Abstract

A 9-year-old neutered male French bulldog presented with gradually progressive tetraparesis. Neurological examination revealed reduced postural reactions in the pelvic limbs and right thoracic limb. Magnetic resonance imaging revealed a 0.9-cm intramedullary mass at the level of the C6 vertebra. The mass was surgically removed via dorsal laminectomy and dorsal midline myelotomy. Histopathological analysis confirmed a hemangioblastoma composed of capillaries and stromal cells. Immunohistochemical analysis revealed that the capillaries were labeled with Factor VIII–related antigen. Stromal cells were labeled with neuron-specific enolase but were not labeled with neurofilaments. Entrapped axons and neurons labeled with neurofilaments were observed within the tumor. This report presents the first case of an intramedullary hemangioblastoma in the cervical spinal cord and provides clinical and pathological insights into hemangioblastomas in dogs.

## 1. Introduction

Hemangioblastomas are rare vascular tumors of the central nervous system in dogs. Various histopathological types of vascular masses, including nonneoplastic lesions, can occur in animals. A hemangioblastoma is classified as a benign neoplasm [1] and is composed of disorganized capillaries and stromal cells. It can be distinguished from capillary hemangiomas by the presence of abundant stromal cells [1].

Hemangioblastomas in dogs are typically solitary lesions [2–7]. In contrast, multiple hemangioblastomas have been reported in humans, most of which are associated with mutations in the von Hippel–Lindau (VHL) gene [8]. Sporadic hemangioblastomas in humans are frequently solitary [8] and are reported to occur more commonly than VHL-associated cases [9, 10]. Furthermore, sporadic hemangioblastomas tend to be larger than those associated with VHL syndrome and are more likely to present with significant neurological clinical signs at the time of diagnosis [8].

Intramedullary hemangioblastomas in dogs have been reported to occur in the cerebrum, brainstem, thoracic spinal cord, and lumbar spinal cord [2–6]. Additionally, an intradural extramedullary hemangioblastoma has been reported to occur in the cervical spinal cord [7]. Until now, its occurrence within the parenchyma of the cervical spinal cord has not been reported. Additionally, information on the surgical treatment for hemangioblastomas is limited.

This report presents the magnetic resonance (MR) imaging (MRI), intraoperative, and pathological findings of an intramedullary hemangioblastoma in the cervical spinal cord of a dog.

## 2. Case Presentation

A 9-year-old neutered male French bulldog was presented with a 3-month history of gradually progressive tetraparesis. Despite proprioceptive ataxia, the dog remained ambulatory. Neurological examination revealed decreased postural reactions in the pelvic and right thoracic limbs, mild hyperreflexia in the pelvic limbs, and mildly reduced superficial nociception in the right thoracic limb. No overt signs of pain were observed. Three weeks later, the neurological condition further deteriorated. The dog presented with nonambulatory tetraparesis and remained predominantly in sternal or lateral recumbency. Postural reactions were absent, and superficial nociception was reduced in all limbs. Spinal reflexes remained mildly hyperactive in the pelvic limbs. However, no obvious abnormalities were observed in the spinal reflexes of the thoracic limbs. Bilateral protrusion of the nictitating membranes was observed. However, no other signs of Horner syndrome, such as ptosis and miosis, were observed. Pain perception remained equivocal throughout the clinical course. The presence of neurological deficits in all four limbs and hyperreflexia in the pelvic limbs suggested a spinal cord lesion involving the C1–5 and C6–T2 segments. Given the normal thoracic limb reflexes, it was not possible to further refine the neurolocalization. A neoplasm originating from the vertebrae, spinal cord, or nerve roots was suspected due to the progressive nature of the clinical signs. Accordingly, computed tomography (CT) and MRI were performed for further evaluation.

**Figure 1:**
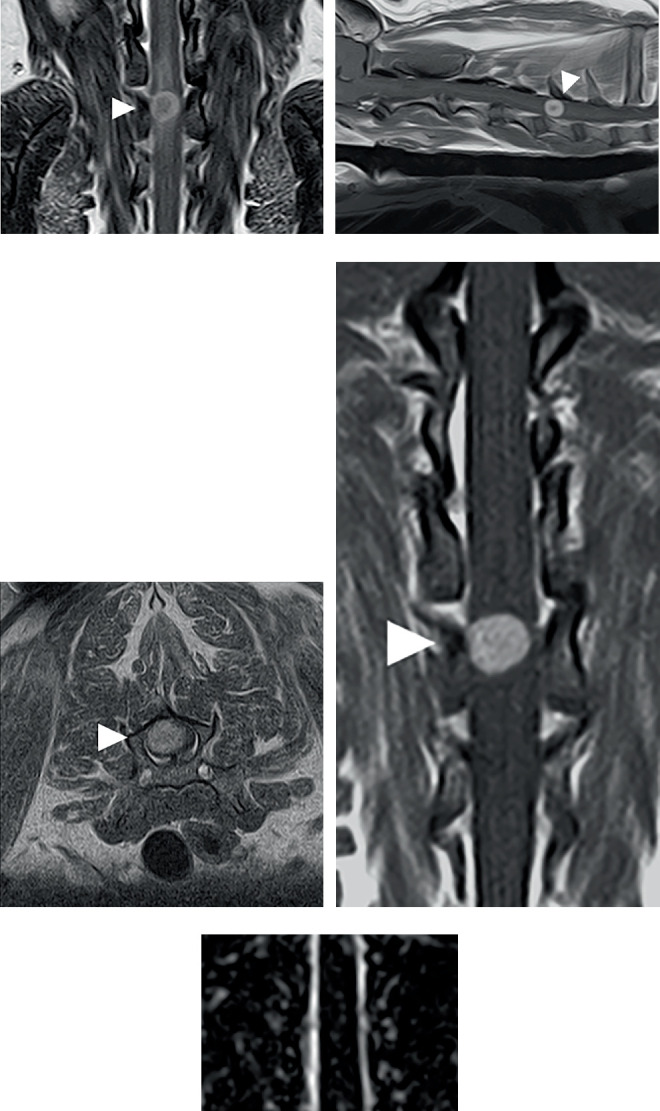
Magnetic resonance imaging (MRI) images of the hemangioblastoma. T2-weighted MRI images in the (a) sagittal, (b) transverse, and (c) dorsal plane. Contrast-enhanced T1-weighted MRI images in the (d) sagittal, (e) transverse, and (f) dorsal plane. (g) MR myelography image in the dorsal plane. White arrowheads refer to the mass.

A CT scan of the entire spine was performed using Aquilion Lightning (Canon Medical Systems). The scanning parameters were 120 kV, 100 mAs, 1-mm slice thickness, 0.5-cm slice spacing, 15 mm/rotation table speed, and 512 × 512 matrix. The CT finding showed vertebral malformation (fusion of the third and fourth cervical vertebrae). MRI of the brain and C1–T2 spinal cord segments was performed using Vantage Elan (Canon Medical Systems) with a knee coil. Technical parameters for T1- and T2-weighted images in the sagittal plane were repetition time (TR) = 350 ms, echo time (TE) = 15 ms, field of view (FOV) = 250 × 200 mm, thickness = 2.5 mm, and spatial resolution = 0.5 × 0.25 mm and TR = 2500 ms, TE = 80 ms, FOV = 250 × 200 mm, thickness = 2.5 mm, and spatial resolution = 0.5 × 0.25 mm, respectively. Technical parameters for T1- and T2-weighted images in the transverse plane were TR = 600 ms, TE = 10 ms, FOV = 80 × 80 mm, thickness = 2 mm, and spatial resolution = 0.3 × 0.3 mm and TR = 3850 ms, TE = 121 ms, FOV = 80 × 80 mm, thickness = 2 mm, and spatial resolution = 0.3 × 0.3 mm, respectively. Technical parameters for T1- and T2-weighted images in the dorsal plane were TR = 350 ms, TE = 15 ms, FOV = 150 × 150 mm, thickness = 2 mm, and spatial resolution = 0.3 × 0.3 mm and TR = 2500 ms, TE = 80 ms, FOV = 150 × 150 mm, thickness = 2 mm, and spatial resolution = 0.3 × 0.3 mm, respectively. T1-weighted imaging was performed in all three planes after intravenous administration of 0.1 mg/kg body weight gadobutrol (Gadovist; Bayer AG) to obtain contrast-enhanced images. Three-dimensional MR myelography was performed by the following technical parameters: single-shot fast spin echo sequence (SS-FSE), TR = 4000 ms, TE = 510 ms, FOV = 200 × 200 mm, thickness = 1 mm, and spatial resolution = 0.25 × 0.25 mm. The MRI findings revealed a 0.9-cm intramedullary mass at the level of the sixth cervical vertebra. The mass exhibited a ring-like hyperintense signal compared to normal spinal cord parenchyma on T2-weighted images (Figures 1a, 1b, and 1c) and an isointense signal compared to normal spinal cord parenchyma on T1-weighted images. Contrast-enhanced T1-weighted images showed that the mass was strongly homogeneously enhanced (Figures 1d, 1e, and 1f). MR myelography showed the absence of the cerebrospinal fluid line at the mass level without curvature (Figure 1g). Based on these findings, an intramedullary mass was suspected.

**Figure 2:**
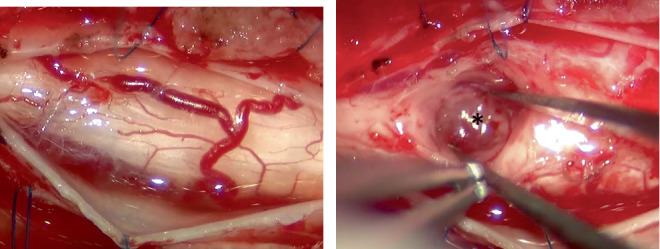
Surgical images of the hemangioblastoma. (a) The spinal cord after dura incision. (b) A dark-red mass (asterisk) in the spinal cord after dorsal midline myelotomy.

A decision was made to remove the mass surgically. The dog was premedicated with subcutaneous administration of atropine (20 *μ*g/kg), followed by intravenous injections of fentanyl (3 *μ*g/kg) and ketamine (0.5 mg/kg). Cefazolin (20 mg/kg) was intravenously administered as a prophylactic antibiotic. General anesthesia was then induced with a slow intravenous injection of 1% propofol until tracheal intubation was achieved. Anesthesia was maintained with sevoflurane inhalation and a constant rate infusion of fentanyl (10 *μ*g/kg/h) and ketamine (0.6 mg/kg/h). A dorsal laminectomy was performed at the C5–6 level to expose the dorsal spinal cord. The dura was incised, and the spinal cord was examined (Figure 2a). However, the tumor was not visible, and no vascular malformations were observed. Mild spinal cord swelling was observed. A dorsal midline myelotomy was then performed to access the intramedullary mass (Figure 2b). The mass was slightly firm, blood-rich, and dark red. The mass was carefully separated from the surrounding tissue under an operating microscope. The mass had mild adhesions, and gross total resection was performed. Wound closure was performed using standard techniques. At the end of surgery, the inhalational anesthetic was discontinued, and extubation was performed after confirming clear spontaneous respiration, palpebral reflex, and laryngeal reflex. Postoperative pain was managed with constant rate infusions of fentanyl (3 *μ*g/kg/h), ketamine (0.6 mg/kg/h), and lidocaine (1.8 mg/kg/h), which were gradually tapered. After recovery from anesthesia, the dog was transferred to the intensive care unit, where physical examination findings were monitored. On Postoperative Day 1, heart rate, blood pressure, respiratory rate, body temperature, and urine output remained generally within normal limits. No pain response was elicited upon palpation of the surgical site. However, the severity of tetraparesis had worsened compared with the preoperative state, and the dog was unable to stand. The superficial nociception was further diminished compared with the preoperative status. Postural reactions and spinal reflexes showed no changes. The respiratory patterns remained normal, and the degree of protrusion of the nictitating membranes remained unchanged compared with the preoperative state. On Postoperative Day 2, physical examination findings remained stable, and neurological examination revealed no changes. The swallowing function was evaluated and was found to be intact. A liquid diet was subsequently provided, and the dog was able to ingest it voluntarily. At 58 h after surgery, the dog suddenly went into cardiac and respiratory arrest and died.

**Figure 3:**
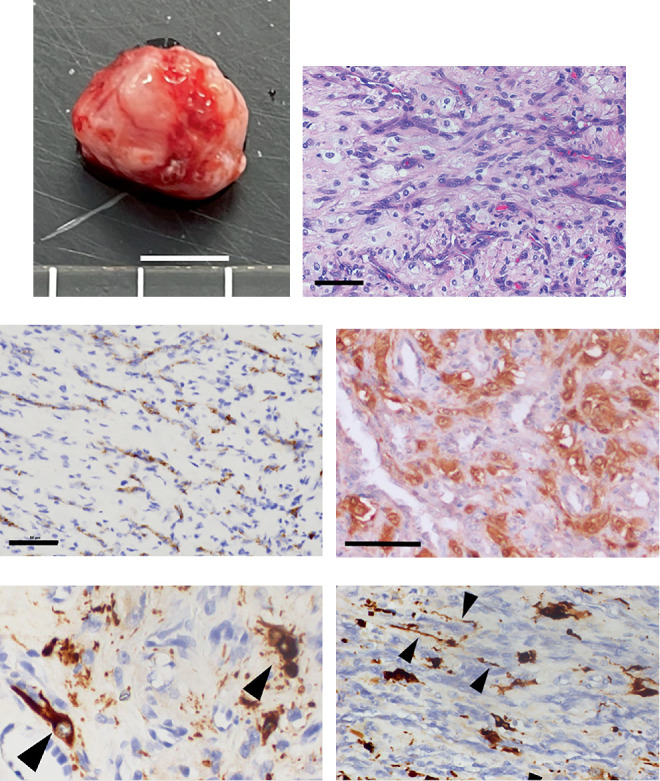
Gross and histopathology images of the hemangioblastoma. (a) The tumor before fixation. Bar = 5 mm. (b) A high-magnification hematoxylin and eosin–stained section showing capillary structures and stromal cells. Bar = 50* μ*m. (c) A high-magnification image showing capillary structures labeled with Factor VIII antigen. Bar = 50* μ*m. (d) A high-magnification image showing stromal cells labeled with neuron-specific enolase. Bar = 50* μ*m. (e) A high-magnification image showing cells with protrusions labeled with neurofilaments (arrowheads) within the tumor. Bar = 25* μ*m. (f) A high-magnification image showing fibers labeled with neurofilaments (arrowheads) between tumor cells. Bar = 50* μ*m.

The resected mass (Figure 3a) was fixed in 10% buffered formalin, embedded in paraffin, sectioned at 5 *μ*m, and stained with H&E for routine histopathological analysis. An abundance of capillary structures was observed within the tumor (Figure 3b). The nuclei of the cells forming these capillary structures were oblong to oval in shape, with indistinct nucleoli. The cytoplasm was amphophilic, resembling that of normal vascular endothelium. Additionally, stromal cells with various shapes were observed between the capillary structures. The nuclei of these stromal cells were round to oval in shape, which were often irregular, with well-defined nucleoli. The cytoplasm of these stromal cells contained small and large vacuoles and was weakly basophilic or amphophilic. The number of mitotic figures was 0–1 per 10 high-power fields (2.37 mm^2^). Immunohistochemical analysis was performed using a polymer-based detection system for further evaluation. The deparaffinized sections were treated with antigen retrieval buffer (BOND Epitope Retrieval ER2 Solution, Leica) for 20 min at 100°C. No antigen retrieval was required for neurofilament staining. After blocking nonspecific binding with 5% skim milk (FUJIFILM Wako) for 20 min at room temperature, the sections were incubated overnight at 4°C with the primary antibodies: rabbit polyclonal anti-von Willebrand factor (1:1000; DAKO) and mouse monoclonal anti-neurofilament (1:100; DAKO). Signal detection was performed using the Histofine Simple Stain MAX-PO (MULTI) polymer reagent (ready-to-use; Nichirei Biosciences) for 60 min at room temperature. Visualization was performed with 3,3⁣′-diaminobenzidine (Nichirei Biosciences), followed by counterstaining with Mayer's hematoxylin. Immunohistochemical staining was similarly performed for the detection of neuron-specific enolase using a rabbit polyclonal anti-neuron-specific enolase antibody (ready-to-use; Nichirei Biosciences) as the primary antibody and the IHC Prep & Detect Kit for rabbit/mouse primary antibody (Proteintech) according to the same protocol. Immunohistochemical analysis revealed that the cells forming the capillary structures were labeled with Factor VIII antigen (Figure 3c). Numerous stromal cells were labeled with neuron-specific enolase (Figure 3d), and most of the stromal cells were not labeled with neurofilaments. However, a few neuron-like cells with dendrites labeled with neurofilaments were sporadically scattered within the tumor (Figure 3e). Additionally, fibers labeled with neurofilaments were observed between the tumor cells (Figure 3f). Based on histopathological and immunohistochemical findings, the mass was diagnosed as a hemangioblastoma.

## 3. Discussion

In humans, the most common site of hemangioblastomas in the central nervous system is the cerebellum, followed by the spinal cord and brainstem [9]. In the spinal cord, the cervical intramedullary region is the most common site of hemangioblastoma development [10]. In dogs, to the best of our knowledge, this is the first report to present a hemangioblastoma in the cervical intramedullary region. Although the dog in the present case was 9 years old, the average age at the onset of hemangioblastomas in dogs has been reported to be relatively young, at 5.2 years (range: 2–9 years) [2–7]. Hemangioblastomas have been reported in a variety of breeds without a marked gender predilection [2–7]. Clinical signs and neurological findings vary depending on the location and size of the hemangioblastoma. However, they generally worsen as the tumor progresses [2, 4, 6].

In our case, the hemangioblastoma exhibited a ring-shaped hyperintensity on T2-weighted MR images, with well-defined margins and an overall homogeneous contrast enhancement. These findings are generally consistent with those of previous reports, although subtle differences can be observed among cases. For example, a thoracic hemangioblastoma has been reported to show ring-like enhancement on contrast-enhanced T1-weighted images [2], whereas a lumbar hemangioblastoma demonstrated homogeneous enhancement [6]. In human medicine, many studies have investigated the correlations between MRI findings and histopathological features. One study showed that hypointensity on T2-weighted images of meningiomas was correlated with a high collagen content [11]. Another study on gliomas showed a correlation between contrast enhancement and histological features, including cellular proliferation and microvascular density [12]. These findings indicate that variations in MRI signal characteristics may at least partly reflect underlying differences in tumor microarchitecture.

Only two cases of spinal cord hemangioblastomas in dogs that were surgically treated have been reported. A hemilaminectomy has been selected in cases of cervical intradural extramedullary and lumbar intramedullary hemangioblastomas [6, 7]. In our case, a dorsal laminectomy was selected to perform dorsal midline myelotomy and resect a large intramedullary tumor. This procedure helped secure a wide FOV and achieve gross total en bloc resection while maintaining hemostasis. The tumor had a very rich blood supply and bled easily from its surface. This is due to the characteristic vascularity of hemangioblastomas. In humans, laminectomy and hemilaminectomy have been reported to be performed for the resection of spinal cord hemangioblastomas in more than half and approximately a quarter of the cases, respectively [10]. Hemilaminectomy is often chosen as a minimally invasive approach for the resection of eccentric intramedullary tumors [13].

Consensus on the optimal myelotomy technique for dogs has not been reached. In humans, posterior midline myelotomy is commonly performed when an intramedullary tumor is centrally located [13–15]. In the present case, the tumor was largely central in location and occupied a significant portion of the spinal cord beyond the midline. Therefore, a posterior (dorsal) midline myelotomy was performed to achieve adequate tumor exposure. A dorsal myelotomy, also known as a dorsal root entry zone myelotomy, is performed when intramedullary tumors are eccentrically located [13, 15]. This approach offers several advantages, including the preservation of the dorsal columns and the use of the nerve rootlets as anatomical landmarks to identify the dorsal lateral sulcus, which serves as the entry point [13]. However, a potential disadvantage of this technique is the increased risk of injury to nearby tracts, including the corticospinal, spinocerebellar, and spinothalamic pathways [13]. An appropriate surgical approach must be selected to ensure adequate tumor exposure while minimizing damage to the spinal cord.

In our case, neurological function deteriorated postoperatively. A previous report of a case of cerebral hemangioblastomas showed a sudden onset of depression and an inability to walk following discharge [5]. In another case of lumbar intramedullary hemangioblastomas, postoperative MRI revealed atrophy of the spinal cord at the surgical site [6]. However, relatively favorable postoperative outcomes, including the ability to ambulate, were reported. A cohort study on human intramedullary tumors showed worsening of neurological function following tumor resection in 32% of cases according to the modified McCormick scale and in 18% of cases according to the American Spinal Injury Association Impairment Scale [16]. Neurological deterioration is a relatively common complication after surgery for intramedullary spinal cord tumors.

Our case died in the early postoperative period, indicating the occurrence of serious complications. A previous study on humans who underwent surgery for intramedullary spinal cord tumors showed that postoperative complications, including cerebrovascular accidents, deep vein thrombosis, delirium, dural leakage, ileus, pneumonia, pulmonary embolism, and urinary tract infections, occurred in 28% of patients [16]. Despite these complications, the postoperative mortality rate in humans has been reported to be low (0.55%–1%) [16, 17]. Additionally, 5-year survival rates in humans have been reported to be 88% for astrocytomas, 90% for ependymomas, and 88% for hemangioblastomas [16]. In contrast, a previous study of intramedullary tumors in dogs reported a median survival duration of 23 days (range: 1–202 days) in cases treated with palliative care, chemotherapy, radiotherapy, surgery, or combinations of these treatments [18]. Given the limited number of reports in veterinary medicine, further accumulation of clinical cases is needed to gain a deeper understanding of the postoperative complications and prognosis of hemangioblastomas in dogs.

Consistent with previous reports of hemangioblastomas in dogs, our case exhibited characteristic capillary structures and abundant stromal cells labeled with neuron-specific enolase. Furthermore, the expression of neurofilaments, a neuronal marker other than neuron-specific enolase, was investigated in stromal cells, and its negativity was confirmed. These findings are consistent with those of previous reports on human hemangioblastomas [19, 20]. Interestingly, in our case, neurofilament-labeled fibers and neuron-like cells were scattered within the tumor, indicating that the hemangioblastoma had entrapped axons and neurons. These findings are similar to those reported in human hemangioblastomas [21].

In conclusion, this case report showed that hemangioblastomas in dogs can develop within the cervical spinal cord parenchyma, can be resected through dorsal laminectomy and median myelotomy, and may involve neural tissues such as axons. Hemangioblastomas in dogs are tumors with limited information available in the literature. Further reports are needed to better understand this tumor.

## Data Availability

Data sharing is not applicable as no datasets were generated in this study. All relevant findings are included in the article.
